# Customized 16S-23S rDNA ITS Amplicon Metagenomics for Acetic Acid Bacteria Species Identification in Vinegars and Kombuchas

**DOI:** 10.3390/microorganisms12051023

**Published:** 2024-05-18

**Authors:** Alja Ribič, Janja Trček

**Affiliations:** 1 Department of Biology, Faculty of Natural Sciences and Mathematics, University of Maribor, SI-2000 Maribor, Slovenia; aljaribic92@gmail.com; 2 Faculty of Chemistry and Chemical Engineering, University of Maribor, SI-2000 Maribor, Slovenia

**Keywords:** *Acetobacter*, *Komagataeibacter*, *Novacetimonas*, ITS metagenomics, microbiota, consortia

## Abstract

Acetic acid bacteria (AAB) are involved in food and beverage production bioprocesses, like those in vinegar and kombucha. They oxidize sugars and alcohols into various metabolites, resulting in the final products’ pleasant taste and aroma. The 16S rDNA amplicon metagenomics using Illumina technology is usually used to follow the microbiological development of these processes. However, the 16S rRNA gene sequences among different species of AAB are very similar, thus not enabling a reliable identification down to the species level but only to the genus. In this study, we have constructed primers for amplifying half of the 16S-23S rRNA gene internal transcribed spacer (ITS) for library construction and further sequencing using Illumina technology. This approach was successfully used to estimate the relative abundance of AAB species in defined consortia. Further application of this method for the analysis of different vinegar and kombucha samples proves it suitable for assessing the relative abundance of AAB species when these bacteria represent a predominant part of a microbial community.

## 1. Introduction

Vinegar and kombucha are microbial products of yeasts, acetic acid bacteria (AAB), and lactic acid bacteria [1,2,3]. These microorganisms transform sugars and alcohols present in various substrates into oxidation products of acidic taste and pleasant aroma [4]. The proportion of individual microbial families, genera, and species varies during the bioprocess due to changes in the chemical composition of the substrate and physical parameters, such as the temperature and amount of dissolved oxygen [5,6].

Semi-synthetic substrates, such as sucrose dissolved in green or black tea, are often used for kombucha production [7]. For vinegar production, consumers prefer natural substrates, such as juice from fruits exposed to restricted treatment in orchards [8,9]. Also, the global orientation to a circular economy tends towards natural substrates that enable the revalorization of organic waste material [10]. The type of substrate determines the growth rate of individual representatives of the microbiota, production efficiency, and the final properties of the product. With the aim to follow the microbiological development of the process, the process has to be monitored quickly and accurately, which is impossible using time-consuming isolation methods that last nine or more days in the case of AAB [11].

Over the past few years, the sequencing of the amplified 16S rRNA gene sequences’ variable regions using Illumina technology has been widely used to profile bacterial communities directly [5,12]. Additionally, Oxford Nanopore technology and PacBio technology have been introduced recently using long-read amplicon sequencing and enabled the entire 16S rRNA gene sequencing or even the entire rRNA operon [13]. The long-read amplicon metagenomic methodology enables higher taxonomic resolution but suffers from robust bioinformatic tools and standardized workflows, the main reason its application has yet to be widely spread [14,15]. Another limitation in 16S rRNA gene sequencing concerns highly conserved 16S rRNA gene sequence in some bacteria, including AAB. Using this target gene, we can only determine changes in the proportion of individual genera of AAB during bioprocesses. Here, we have developed a methodological approach that enables direct species identification based on partial ITS amplicon metagenomics in microbiota with a predominant AAB population.

## 2. Materials and Methods

### 2.1. Construction of Oligonucletides for ITS Metagenomics

The 16S-23S rDNA internal transcribed spacer (ITS) of *Acetobacter*-, *Novacetimonas*-, and *Komagataeibacter*-type strains were aligned. The most conserved region representing the middle part of 16S-23S rDNA ITS and coding for tRNA^Ala^ was used for the construction of two primers: tAla2Rev (5′-GCAGGTGCTCTCCCAGC-3′; position 294 to 278 on 16S-23S rDNA ITS *Gluconacetobacter entanii* LTH 4560^T^, acc. no. HE861939) and tAla2Fw (5′-CCCGTCYGYCTCCACCA-3′; position 323 to 339 on 16S-23S rDNA ITS *G. entanii* LTH 4560^T^, acc. no. HE861939). Primer tAla2Rev in combination with primer SpaFw (5′-TGCGGCTGGATCACCTC-3′; position 1522-1549 on 16S rDNA *Escherichia coli* numbering) amplifies the first part of the ITS region, and primer tAla2Fw in combination with primer SpaRev (5′-GTGCCAAGGCATCCACC-3′; position 38-22 on 23S rDNA *E. coli* numbering) amplifies the second part of the ITS region. Each of the products is approximately 250 bp large and thus suitable for Illumina sequencing technology. The primers SpaFw and SpaRev have been previously described by Trček and Teuber [16] and are routinely used for the species identification of AAB [17,18]. The primers were synthesized at Microsyth AG (Balgach, Switzerland).

### 2.2. Preparaton of Synthetic Acetic Acid Bacteria Microbiota

The bacterial strains used in this study were as follows: *Gluconacetobacter entanii* AV429, *Komagataeibacter kakiaceti* LMG 26206^T^, *Komagataeibacter oboediens* AV371, *Komagataeibacter saccharivorans* BJK_3A, *Komagataeibacter swingsii* LMG 22125^T^, and *Novacetimonas hansenii* LMG 1527^T^. The strains have been revitalized from −80 °C on the RAE medium (glucose 40 g/L, peptone 10 g/L, yeast extract 10 g/L, citric acid 1.37 g/L, Na_2_HPO_4_ × 2 H_2_O 3.38 g/L, agar 10 g/L) containing glacial acetic acid (1 vol%) and absolute ethanol (1 vol%) with incubation at 30 °C and 92–96% relative air humidity for 3 days.

To obtain the AAB community of known composition, each of the strains was grown until it reached the exponential growth rate (absorbance at 600 nm 0.8–1.0) and then mixed in equal proportions to mimic a natural AAB microbiota. Each bacteria culture was also spread on the agar plates to determine the cell numbers after counting CFU/mL. The cell’s size of different species was also evaluated under microscope using 1000-fold magnification.

### 2.3. ITS and Full-Length 16S rDNA Amplicon Metagenomic Analysis

The samples used for metagenomics representing different vinegars and kombuchas are listed in Table 1. After collection, the samples were transported to the laboratory and directly processed. If the bacteria were suspended in the sample, the cells were separated from the vinegar by centrifugation (10,000 rpm, 10 min) and washed with 0.85% NaCl. If bacteria were embedded into the biofilm, approximately 0.35 g of biofilm was cut into small particles by a sterile scalpel, washed with 0.85% NaCl, and proceeded to DNA isolation. Two commercial kits have been tested for DNA isolation from the AAB community: NucleoSpin Tissue (Macherey-Nagel, Düren, Germany) and GeneJet Genomic DNA Purification Kit (Thermo Scientific, Waltham, MA, USA). The protocol for the isolation of DNA from Gram negative bacteria has been used.

The samples of DNA were sent to Novogene (Cambridge, United Kingdom) and proceeded to quality control and then to libraries preparation using customized primers described under 2.1 for ITS metagenomics and standard primers 16SFw (5′-AGAGTTTGATCCTGGCTCAG-3′) and 16SRev (5′-GNTACCTTGTTACGACTT-3′) for full-length 16S rDNA metagenomics (Novogene, personal communication). The ITS libraries were sequenced on an Illumina NovaSeq 6000 platform by generating 250 bp paired-end reads and 30 K raw tags data output. The full-length 16S rDNA libraries were sequenced with PacBio Sequel II technology using the HiFi/CCS mode with the generation of 0.01 M clean reads per sample.

Sequences were analyzed at the Novogene as follows. Paired-end reads were assigned to samples based on their unique barcode and truncated by cutting off the barcode and primer sequence. Paired-end reads were merged, and quality filtering on the raw tags were performed to obtain the high-quality clean tags by utilising QIIME2 (v2020.6) [19], with the plugins VSEARCH [20]. The tags were compared with the reference database (Gold database, http://drive5.com/uchime/uchime_download.html; accessed on 10 January 2024) using UCHIME algorithm to detect chimera sequences, and then the chimera sequences were removed [21]. Minimal 96% all bases were above Q30 (base call accuracy of 99.9%) in Illumina NovaSeq 6000 and PacBio Sequel II platform.

In the case of ITS metagenomics, sequence analysis was performed using UPARSE software (v7.0.1001) [22] by clustering the sequences to operational taxonomic units (OTUs) with ≥97% similarity. For full-length 16S rDNA amplicon metagenomics, the amplicon sequence variants (ASVs) were denoised with a 100% similarity using the DADA2 algorithm in QIIME2 software [19]. The Blast algorithm at NCBI (https://blast.ncbi.nlm.nih.gov/Blast.cgi?PROGRAM=blastn&PAGE_TYPE=BlastSearch&LINK_LOC=blasthome; accessed on 20 February 2024) was further used to analyze the sequences with ≥0.01% relative abundance. 

### 2.4. Acidity and pH of the Samples

The acidity of the samples was determined by titrating the samples with 0.1 M NaOH in the presence of phenolphthalein and by measuring the pH.

## 3. Results and Discussion

### 3.1. Optimization of ITS Amplicon Metagenomics for AAB Species Identification

**Figure 1:**
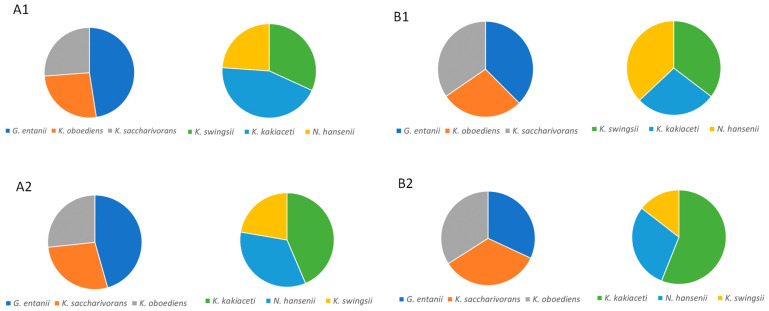
Comparison of relative species abundance in two defined AAB consortia. Letters (**A**) and (**B**) mark results obtained by commercial kits GeneJet and NucleoSpin Tissue for DNA isolation (see Section 2.3), respectively. Number **1** denotes the application of primers SpaFw and tAla2Rev, and number **2** represents the primers tAla2Fw and SpaRev (see Section 2.3).

We first tested two primers (see Section 2.1) for species identification in the consortia composed of defined AAB species. Besides two sets of primers, we also analyzed two commercial kits for DNA isolation. The consortia were composed of three species mixed in an equal proportion as described in Section 2.2 and used for DNA isolation. The results in Figure 1 show a comparison of the relative abundance of each species in two types of consortia. The primers SpaFw and tAla2Rev amplifying the first part of the ITS region and commercial kit NucleoSpin Tissue produced results that strongly support the species’ relative abundance, as expected from the preparation of the species-defined consortia. Some deviation from the anticipated relative abundance may result from differences in cells’ sizes, which affects the amount of material used for DNA isolation. These primers and the kit for DNA isolation were selected for further experiments.

### 3.2. Comparison between Full-Length 16S rDNA and ITS Amplicon Metagenomics

**Figure 2:**
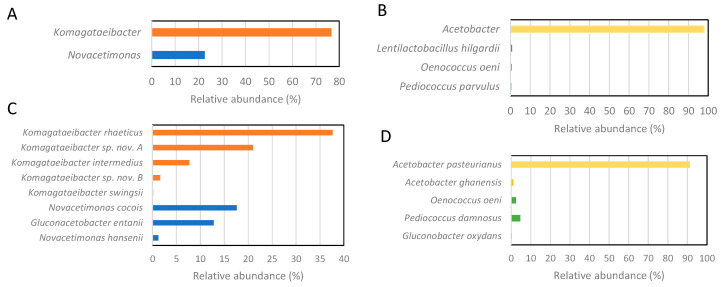
Comparison of relative abundance of AAB detected by 16S rDNA and 16S-23S rDNA ITS amplicon metagenomics. Results on bacteria identification using 16S rDNA amplicon metagenomics of apple cider and wine vinegars are presented under (**A**,**B**), respectively. The results based on 16S-23S rDNA ITS amplicon metagenomics of the samples taken from the same bioprocess are presented under (**C**,**D**) for apple cider and wine vinegars, respectively.

To compare the taxonomic discriminatory power of full-length 16S rDNA and partial ITS amplicon metagenomics for the identification of AAB species, we performed both analyses from the same apple cider vinegar and wine vinegar samples. The results in Figure 2 show that although a full-length 16S rRNA gene is used for library construction, this taxonomic marker enables reliable identification only to the genus level of AAB. In contrast, the ITS region, although we used only half of its length due to the technical limitations of applied Illumina technology, can identify the species of AAB. However, the ITS metagenomics also shows the following restriction: the primers also bind to the DNA of lactic acid bacteria, if present in a sample (Figure 2D), although not necessarily with a comparable specificity as primers for 16S rRNA gene (Figure 2B). This drawback may be improved by sequencing the entire 16S-23S rDNA ITS using customized amplicon long-read sequencing when commercially available.

### 3.3. Comparison of AAB Species Structure among Different Samples

**Figure 3:**
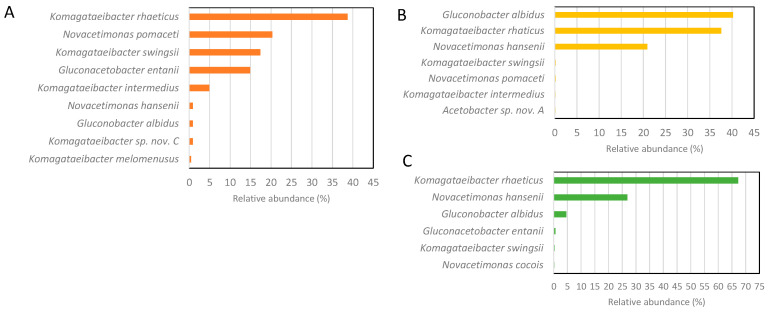
The relative abundance of AAB species determined by 16S-23S rDNA ITS amplicon metagenomics in elderflower vinegar EldVi (**A**) and kombucha pellicles of KomOmaK2 (**B**) and KomOmaH2 (**C**). The kombucha samples presented under B and C were taken from different stages of their production.

The method presented above was then used to analyze the real samples of vinegars and kombuchas. The results presented in Figure 3 show the microbiota structure of the biofilm layer floating at the top of vessels for the static production of elderflower vinegar and two types of kombuchas.

The AAB pellicle of the elderflower vinegar produced from fermented elderflower syrup has not been analyzed so far. This is the first report showing *Komagataeibacter rhaeticus* (38.7%) as the predominant species in this type of vinegar, followed by *Novacetimonas pomaceti* (20.3%), *Komagataeibacter swingsii* (17.4%), *Gluconacetobacter entanii* (14.9%), and *Komagataeibacter intermedius* (4.9%). The other identified species, *Novacetimonas hansenii*, *Gluconobacter albidus*, *Komagataeibacter melomenusus*, and a potentially novel *Komagataeibacter* species, were present below 1%. The species richness in this type of vinegar is comparable to a recent study on 23 kombucha microbiota collected in the USA [3], where the starters contain, on average, five AAB species with a relative abundance > 1%.

The microbial diversity of kombucha tea fermentation has been analyzed in some studies using different sequencing technologies over the last few years. The first culture-independent study of five kombuchas’ pellicles from Canada, Ireland, and the United Kingdom showed the *Gluconacetobacter* genus (later reclassified into *Komagataeibacter*) as the major genus in all samples. Since the method was based on the metagenomics of the amplified partial 16S rRNA gene, the sequences enabled identification only to the genus level [23]. A more precise sequencing approach, shotgun metagenomic, revealed *Komagataeibacter rhaeticus* as the most common species in kombucha samples from across the USA and in two analyzed Turkish kombucha samples [3,24,25]. The second most common species, with an 8% relative abundance, was *Komagataeibacter xylinus* [25]. Kombucha samples analyzed in our work identify *K. rhaeticus* as a significant AAB community species. However, it was a predominant species in only one of both analyzed samples. A possible explanation is the different kombucha bacteria community composition in various stages of kombucha production, which also reflects a difference in the pH of both analyzed samples (KomOmaK2, KomOmaH2).

**Figure 4:**
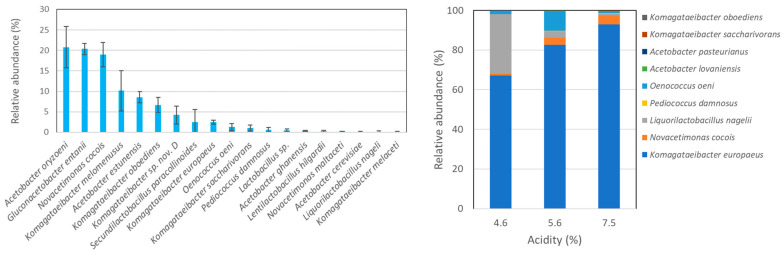
Relative abundance of AAB species in two different submerged processes for apple cider vinegar production. The left panel represents the process ApViSR, and the right panel the process ApViSB (see Table 1).

Static production of vinegar and kombucha usually proceeds in lower quantities and for home use only. In contrast, submerged production is the most widely used technological approach for quick and large amounts of vinegar production at the industrial levels. Here, we have analyzed samples taken from a submerged bioreactor for apple cider vinegar production (Figure 4).

Apple cider vinegar produced in submerged bioreactors differ substantially in AAB species richness (Figure 4). Considering the species with over 1% relative abundance, the sample ApViSR contains nine species representing three different AAB genera. The sample ApViSB has only one major species, i.e., *K. europaeus*. These are the first data on apple cider vinegar microbiota species composition analyzed by amplicon metagenomics. The substantially higher species richness of the sample ApViSR may be attributed to the fact that this type of vinegar was produced from organic apples, which possess more heterogeneous bacterial microbiota in comparison to the apples from conventional orchards [26], that were substrate for the sample ApViSB. Previously, a microbiota composition during organic apple cider vinegar production in an industrial submerged bioreactor has been evaluated by denaturing high-pressure liquid chromatography (DHPLC) and Illumina MiSeq sequencing [5]. The AAB consortium comprised *Acetobacter* and *Komagataeibacter* and a minor genus *Gluconobacter*, even at an acidity of 5%. 

## 4. Conclusions

The method presented in this work for identifying the AAB species directly from microbial consortia is a further step in developing molecular approaches for the refined identification of this food-grade bacteria without culturing. The approach may be further improved using NGS technology that allows amplicon metagenomics with customized primers and longer reads. Here, the method was successfully used for the species identification of vinegar and kombucha microbiota but may be extended to other products, such as kefir, nata-de-coco, beer, and wine, and industrial microbial processes for acids, sugars, and vitamin production by AAB starters.

## Figures and Tables

**Table 1:** List of samples used for metagenomic analysis and their basic characteristics.

Type of Sample	Sample Designation	Type of Production	Origin	Date of Sampling	Acidity (%)	pH
Apple cider vinegar	ApViC	Static production in 600 L vessel	Farm Čarička, Razkrižje, Slovenia	March, 2023	6.1	3.2
Elderflower vinegar	EldVi	Static production in 600 L vessel	Farm Čarička, Razkrižje, Slovenia	March, 2023	2.9	2.2
Kombucha	KomOmaK2	Static production in 25 L vessel	Homemade, Lovrenc na Pohorju, Slovenia	April, 2023	2.0	2.7
Kombucha	KomOmaH2	Static production in 2 L vessel	Homemade, Lovrenc na Pohorju, Slovenia	July, 2023	2.1	3.3
Apple cider vinegar	ApViSR	Submerged production in 8000 L bioreactor	Šampionka Renče, Volčja Draga, Slovenia	January, 2014	4.5	3.1
Apple cider vinegar	ApViSB	Submerged production in 300 L bioreactor	Slovenska Bistrica, Slovenia	August, 2023	4.6–7.5	3.0–3.2

## Data Availability

The raw data supporting the conclusions of this article will be made available by the authors on request.
